# Role of *Arctium lappa* L. Root (Burdock) Extract in CFA-induced Arthritis Rat Model: Impact on Antioxidant Activity and Inflammation

**DOI:** 10.5812/ijpr-162189

**Published:** 2025-07-08

**Authors:** Mohammad Hosein Farzaei, Arezoo Moini Jazani, Mina Salimi, Sahar Shafiei, Hosna Khazaei, Mohammad Hashemnia, Mohammad Mehdi Gravandi, Ramin Nasimi Doost Azgomi

**Affiliations:** 1 Traditional Medicine and Hydrotherapy Research Center, Ardabil University of Medical Sciences, Ardabil, Iran; 2 Pharmaceutical Sciences Research Center, Health Institute, Kermanshah University of Medical Sciences, Kermanshah, Iran; 3 Student Research Committee, Faculty of Pharmacy, Kermanshah University of Medical Sciences, Kermanshah, Iran; 4 Department of Pathobiology, Faculty of Veterinary Medicine, Razi University, Kermanshah, Iran

**Keywords:** *Arctium lappa*, Rheumatoid Arthritis, Oxidative Stress, Inflammation, Complete Freund's Adjuvant (CFA)

## Abstract

**Background:**

*Arctium lappa* L. is a well-known medicinal herb recognized for its anti-inflammatory and antioxidant properties, which have been shown to be beneficial in the treatment of various diseases.

**Objectives:**

The present study aimed to explore the protective effects of the hydroalcoholic extract of *A. lappa* root in complete Freund’s adjuvant (CFA)-induced arthritis in rats.

**Methods:**

Arthritis was induced in Sprague-Dawley rats through a single subcutaneous CFA injection into the right hind paws. After induction, the animals were orally administered *A. lappa* at doses of 200 and 400 mg/kg, or prednisolone (as a reference drug), for a duration of 30 days. Blood and ankle joint samples were collected for analysis. The anti-arthritic effects were assessed through nociceptive behavioral tests, paw edema, body weight measurements, serum concentrations of tumor necrosis factor-α (TNF-α), nitric oxide (NO), glutathione (GSH), superoxide dismutase (SOD) activity, and histopathological evaluations.

**Results:**

The *A. lappa* root extract significantly improved body weight and reduced thermal hyperalgesia, as well as decreased paw edema in a dose-dependent manner (P < 0.001). Furthermore, *A. lappa* reduced inflammatory cytokines and increased antioxidant defenses in the serum of all treated rats (P < 0.05 to P < 0.001). Histologically, *A. lappa* notably restored the ankle joint architecture compared to untreated arthritic rats (P < 0.05 to P < 0.001).

**Conclusions:**

The results suggest that *A. lappa* has significant anti-arthritic potential by reducing inflammatory cytokines and enhancing serum antioxidant levels, which supports its traditional use in the management of joint diseases.

## 1. Background

Rheumatoid arthritis (RA) is a widespread autoimmune condition marked by persistent inflammation, impacting around 0.5% to 1% of the global population (1). The cardinal features of uncontrolled RA include synovial hyperplasia, joint swelling, and erosive cartilage destruction, with or without extra-articular involvement (2, 3). Rheumatoid arthritis predominantly affects the elderly population and, without timely intervention, can result in significant disability, reduced quality of life, and even mortality (4). The exact etiology of RA is not clearly understood. It is assumed that interplay among genetic factors and the environment may lead to systemic immune dysregulation, resulting in chronic persistent synovial inflammation (5). The disease is associated with enhanced pro-inflammatory cytokine expression [e.g., interleukins (IL)-1β, IL-6, and tumor necrosis factor (TNF-α)] that play pivotal roles in development and progression (6, 7). Moreover, the participation of oxidative stress in the aggravation of chronic inflammatory joint disease, such as RA, has been well investigated (8). It has been known that free radicals can function as a secondary messenger in immunological cellular responses and cause joint degeneration in RA (9, 10). Several experimental and clinical investigations demonstrated elevated lipid peroxidation, reactive oxygen species (ROS) production, and impaired antioxidant systems (10-12). At present, the commonly utilized medications against RA comprise analgesics, NSAIDs, glucocorticoids, and disease-modifying antirheumatic drugs (DMARDs) (13). However, researchers’ focus has shifted towards complementary and alternative herbal medicines that are safer and less toxic.

Herbal products have always played a crucial role in discovering new therapeutic agents. *Arctium lappa* L., commonly called burdock, is a perennial herb of the Asteraceae family and primarily grows in Asia, Europe, and North America (14). *Arctium lappa* root extracts contain several compounds, including inulin, volatile oils, tannin, resin, polysaccharides, minerals, chlorogenic acid, caffeic acid, quercetin, arctigenin, arctiin, and vitamins (15). It has been reported that the extract of *A. lappa* roots is utilized as an antimicrobial, anti-diabetic, antioxidant, gastro-defensive, analgesic, and anti-hypertensive agent (16, 17).

## 2. Objectives

Recent investigations have demonstrated the immunosuppressive, anti-tumor, and anti-inflammatory impacts of *A. lappa* root extracts (18, 19). However, its protective effect against RA has not been examined. This investigation aimed to assess the impact of *A. lappa* root extract on nociceptive behavior, oxidative stress, and inflammatory markers in a complete Freund’s adjuvant (CFA)-induced arthritic rat (CFA-RA) model.

## 3. Methods

### 3.1. Preparation of Hydroalcoholic Extract of Arctium lappa L. Roots

In July 2021, fresh *A. lappa* L. roots were obtained from a local herbal market in Kermanshah, Iran. The roots were air-dried at room temperature in the shade and then ground into powder using a herb grinder. For the preparation of the hydroalcoholic extract, approximately 200 g of root powder was soaked in 1 L of 80% (v/v) ethanol for 72 hours. After filtration, the supernatant was evaporated to dryness using a rotary evaporator (Heidolph-Laborota 4001, Germany) under reduced pressure. The dried extract was then reconstituted in normal saline and stored at 4°C for future use (20).

### 3.2. Standardization of Plant Extract

For the quantitative measurement of chlorogenic acid (C3878, Sigma, USA), high-performance liquid chromatography (HPLC) was employed. Sample preparation was carried out as follows: Dry extract (0.5 g) was placed in a 100 mL volumetric flask, and 80 mL of 70% ethanol was added. The resulting solution was treated in an ultrasonic bath for 15 minutes. After cooling, the volume of the solution was adjusted to 100 mL with 70% ethanol and filtered through a 0.45 μm filter. The prepared samples were stored in the refrigerator at 5°C until ready for injection. For liquid products, the sample was diluted 1:5 with the mobile phase after filtration before being injected into the HPLC instrument.

### 3.3. Instrumental Conditions

The analysis was performed using a Knauer HPLC system. The column used was an Eclipse-XDB-C18, with dimensions of 25 cm × 4.6 cm × 5 µm. The pump was a Knauer K-1001, and the detector was a Knauer UV K-2501 set at a wavelength of 330 nm. The flow rate was set to 1.5 mL/min, and the column temperature was maintained at 35 ± 1°C. The mobile phase consisted of two components: Phase A (a solution of phosphoric acid and water in a 1:999 v/v ratio) and phase B (acetonitrile).

### 3.4. Animals

The study utilized 25 male Sprague-Dawley rats, aged between 8 and 10 weeks, with a weight range of 180 to 200 g. The animals were obtained from the Faculty of Pharmacy, Lorestan University of Medical Sciences, Iran. The rats were allowed to acclimate for one week in a controlled environment, where the temperature was maintained at 22 ± 3°C, with a humidity level of 60 - 70% and a 12-hour light/dark cycle. The rats had free access to water and were fed a standard pellet diet. All experimental procedures were approved by the Ethics Committee of Ardabil University of Medical Sciences (IR.ARUMS.REC.1400.138) and followed the NIH guidelines (Publication No. 82-23, revised 1985).

### 3.5. Research Design

The rats were allocated into five groups, each consisting of five animals (5 rats per group), as outlined below:

- Cohort I: Normal control (NC)

- Cohort II: CFA-triggered arthritic (RA)

- Cohort III: RA + prednisolone (P, 10 mg/kg/day, orally)

- Cohort IV: RA + *A. lappa* (200 mg/kg/day, orally)

- Cohort V: RA + *A. lappa* (400 mg/kg/day, orally)

On day 1, arthritis was induced by a single subcutaneous injection of 0.1 mL CFA (Sigma Aldrich, USA), containing 10 mg/mL of heat-killed Mycobacterium tuberculosis in mineral oil, into the right hind paw footpad of rats under light isoflurane anesthesia (21). Six days post-induction, cohorts I and II (NC and RA control) were administered saline (as the extract solvent) for 30 days. Cohort III received prednisolone (10 mg/kg/day) (22) orally as a reference drug. Cohorts IV and V were given the hydroalcoholic extract of *A. lappa* (200 mg/kg and 400 mg/kg, respectively) orally, once daily for 30 days (23, 24).

### 3.6. Paw Edema Assessment

The paw volume of rats was monitored weekly from day 0 (before CFA injection) to day 35 of arthritis induction using a plethysmometer (Ugo Basile, Italy) (25).

### 3.7. Thermal Hyperalgesia (Hot Plate Test)

Thermal hyperalgesia was evaluated using the hotplate test, following established protocols (26). Briefly, the animals were acclimated to the testing room for 15 minutes. Each rat was then placed on a hotplate (Harvard Apparatus, USA) set at 52 ± 0.5°C, and the latency to pain responses (e.g., paw jumping or licking, indicating nociceptive threshold) was measured. A maximum cut-off time of 60 seconds was set to avoid tissue injury. Each rat underwent the test three times with 5-minute intervals between trials, and the average response time was determined.

### 3.8. Weight Evaluation

The body weight of the rats was recorded starting from day 0 and subsequently on days 4, 8, 12, 16, 20, 24, 28, 32, and 35 post-CFA injection using a digital scale.

### 3.9. Sampling

On the 36th day, the rats were sedated through intraperitoneal administration of xylazine (10 mg/kg) and ketamine (100 mg/kg). Blood was drawn from the tail vein and centrifuged at 3000 × rpm for 10 minutes at 4°C to separate the serum for further analysis. Afterward, the animals were euthanized, and their right ankle joints were surgically removed and fixed in 10% formalin for histopathological evaluation.

### 3.10. Biochemical Assays

Serum TNF-α and nitric oxide (NO) concentrations were measured using an ELISA kit (Kiazist, Iran). Additionally, serum glutathione (GSH) concentrations and superoxide dismutase (SOD) activity were quantified with a commercial ELISA kit (Kiazist, Iran) according to the manufacturer’s protocol.

### 3.11. Histopathological Changes

At the end of the experiment, the ankle joints from the euthanized rats were dissected and fixed in 10% formalin for 24 hours. The joints were then decalcified using 5% nitric acid, embedded in paraffin, and sectioned into 5 μm slices. These slices were stained with hematoxylin and eosin (H&E) and examined under a light microscope (Nikon E600; Nikon, Tokyo, Japan) (27). The histopathological evaluation focused on parameters such as synovial hyperplasia, bone destruction, vascular proliferation, and inflammatory cell infiltration. The scoring criteria for these parameters followed the methodologies outlined by Schramm et al. (28) and Wang et al. (29).

### 3.12. Statistical Analysis

Data were expressed as mean ± SEM and analyzed using GraphPad Prism software (version 8; GraphPad Inc., USA). Statistical significance among cohorts was determined with one-way or two-way repeated measures ANOVA, followed by Tukey’s post-hoc assessment. A P < 0.05 was considered statistically significant.

## 4. Results

### 4.1. High-performance Liquid Chromatography Analysis of Arctium lappa L. Root Extract

**Figure 1.**
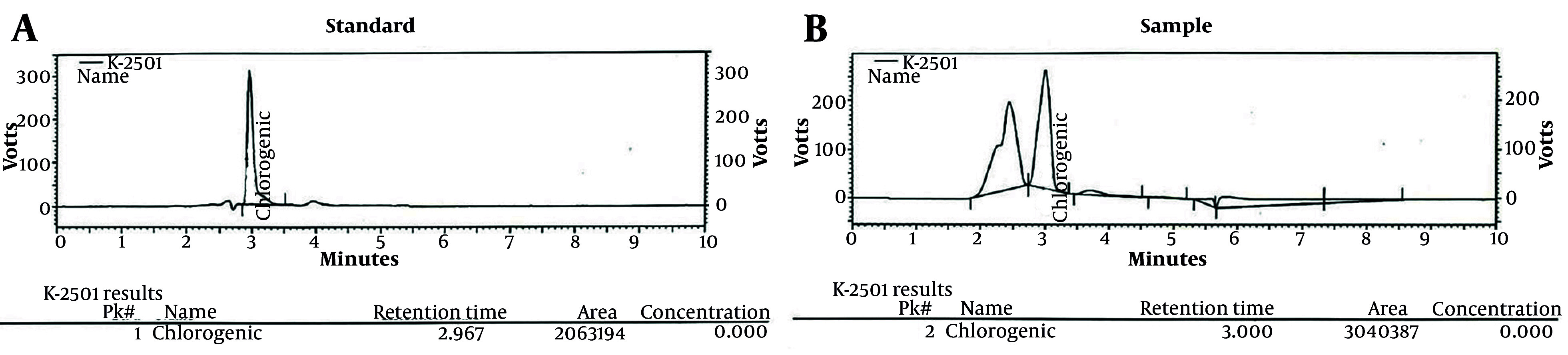
UV on A, line and high-performance liquid chromatography (HPLC) chromatogram of standard (chlorogenic acid) and B, chlorogenic acid existing in the *Arctium lappa* L.

According to the standard and sample graphs, as well as the location of the peaks in both graphs, the retention times were found to be identical (RT standard: 2.967, RT sample: 3), as shown in Figure 1A and B. Moreover, using equation x, the amount of chlorogenic acid in the extract sample was calculated to be 29.10 mg/g.


$$\frac{A_{3}\times C_{2}\times 100\times 0.695}{A_{2}\times C_{1}}$$

A3: The area under the chlorogenic or chicoric acid peak in the sample chromatogram

A2: The area under the chlorogenic or chicoric acid peak in the reference standard chromatogram

C2: The reference concentration of a standard in mg/mL

C1: The concentration of a test sample in mg/mL

### 4.2. Impact of Arctium lappa Root Extract on the Body Weight of Rheumatoid Arthritis Rats

**Figure 2.**
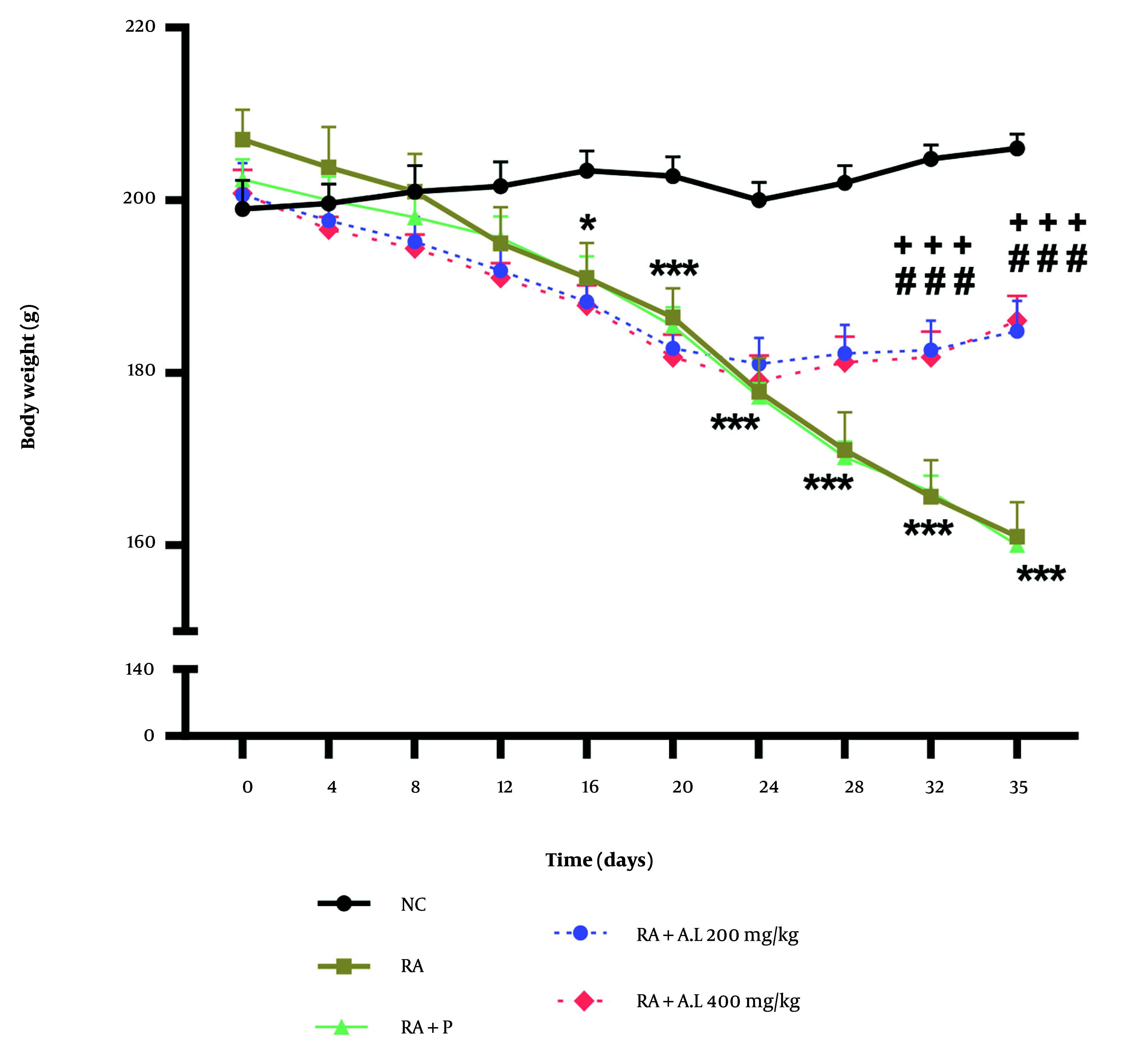
Effect of *Arctium lappa* extract on body weight in RA-induced rats. Data are presented as mean ± SEM (n = 5). Significant differences were tested by two-way repeated measures ANOVA followed by Tukey’s post-hoc test; * P < 0.05, *** P < 0.001 vs. NC group. ### P < 0.001 vs. RA group. +++ P < 0.001 vs. RA + P group. (Abbreviations: NC, normal control; RA, rheumatoid arthritis; P, prednisolone; AL: *Arctium lappa*).

The two-way repeated measures ANOVA indicated significant variations in body weight based on time [F (9, 200) = 42.69, P < 0.0001], intervention [F (4, 200) = 56.86, P < 0.0001], and a notable intervention by time interaction [F (36, 200) = 7.195, P < 0.0001]. As shown in Figure 2, the body weight of the RA cohort decreased markedly relative to the NC cohort following RA induction, with significant variations recorded from days 16 to 35 (P < 0.05 to P < 0.001). However, administering *A. lappa *extract at doses of 200 and 400 mg/kg to RA rats resulted in a significant enhancement in body weight on days 32 and 35 relative to the arthritis control cohort (P < 0.001 for both) (Figure 2). The findings suggest that *A. lappa* (200 and 400 mg/kg) was more effective than prednisolone in preventing weight loss in arthritic rats on days 32 and 35 of the experiment (P < 0.001 for both) (Figure 2). 

### 4.3. Impact of Arctium lappa Root Extract on the Hind Paw Volume of Rheumatoid Arthritis Rats

**Figure 3.**
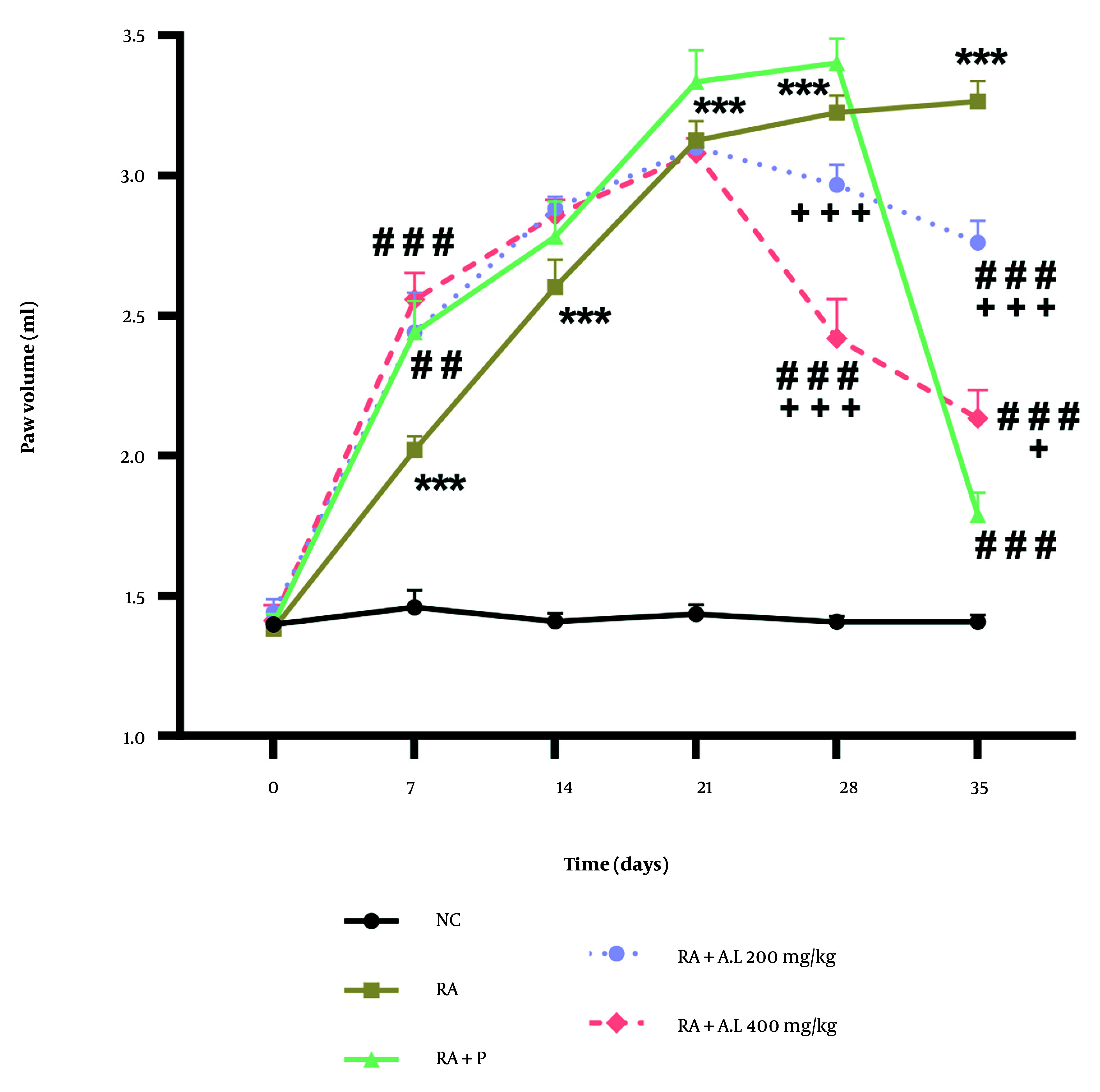
Effect of *Arctium lappa* extract on paw volume in RA-induced rats. Data are presented as mean ± SEM (n = 5). Significant differences were tested by two-way repeated measures ANOVA followed by Tukey’s post-hoc test; *** P < 0.001 vs. NC group. ## P < 0.01, ### P < 0.001 vs. RA group. + P < 0.05, +++ P < 0.001 vs. RA + P group. (Abbreviations: NC, normal control; RA, rheumatoid arthritis; P, prednisolone; AL, *Arctium lappa*).

The two-way repeated measures ANOVA demonstrated significant main effects over time [F (5, 120) = 217.5, P < 0.0001] and intervention [F (4, 120) = 262.6, P < 0.0001], as well as a significant interaction between intervention and time [F (20, 120) = 30.09, P < 0.0001]. The post-hoc Tukey test showed that the cohorts treated with the hydroalcoholic extract (200 and 400 mg/kg) and the RA + P cohorts significantly mitigated CFA-triggered edema on the 28th, and 35th days of intervention (P < 0.001) (Figure 3). Additionally, there were significant differences in paw volume between the RA + *A. lappa* cohorts (200 and 400 mg/kg) and the RA + P group on days 28 and 35 of the experiment (P < 0.05 to P < 0.001) (Figure 3). 

### 4.4. Impact of Arctium lappa Root Extract on Thermal Hyperalgesia of Rheumatoid Arthritis Rats

**Figure 4.**
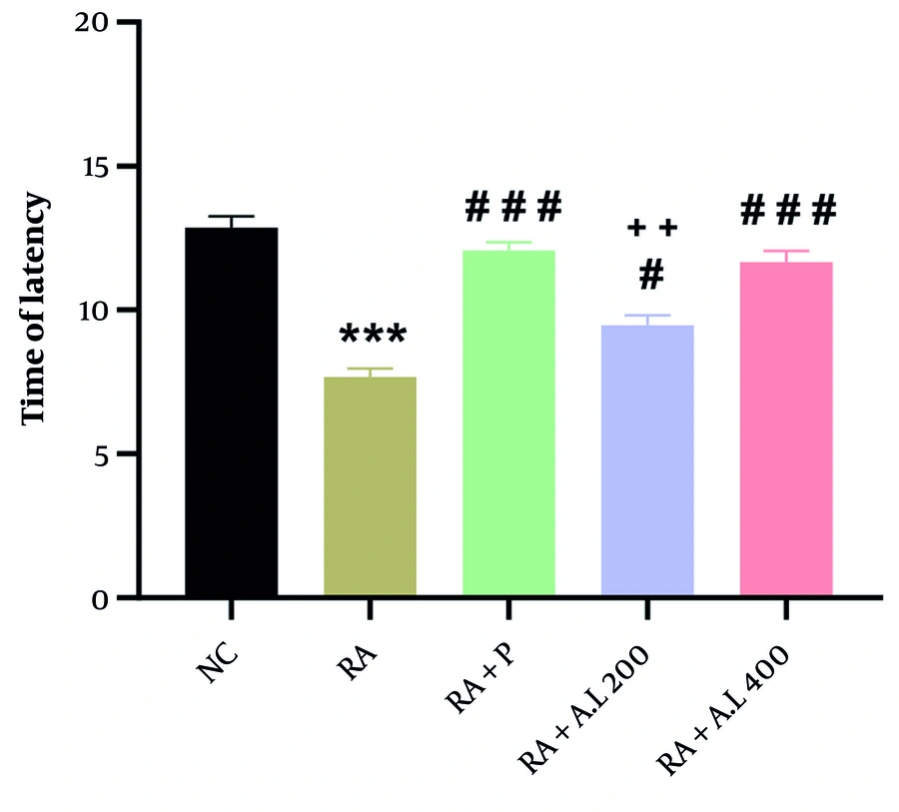
Effect of *Arctium lappa* extract on thermal hyperalgesia in RA-induced rats. Data are presented as mean ± SEM (n = 5). Significant differences were tested by one-way ANOVA followed by Tukey’s post-hoc test; *** P < 0.001 vs. NC group. # P < 0.05, ### P < 0.001 vs. RA group. ++ P < 0 .01, vs. RA + P group. (Abbreviations: NC, normal control; RA, rheumatoid arthritis; P, prednisolone; AL, *Arctium lappa*).

A statistically significant difference among the groups was identified through one-way ANOVA [F (4, 20) = 38.94, P < 0.0001, Figure 4]. The CFA-injected rats exhibited a significant decrease in the thermal hyperalgesia threshold compared to the NC cohort (P < 0.001). However, the cohorts treated with *A. lappa* (200 and 400 mg/kg) and the RA + P cohort showed a notable increase in the thermal hyperalgesia threshold compared to the RA cohort (P < 0.05 for 200 mg/kg; P < 0.001 for 400 mg/kg and prednisolone), in a dose-dependent manner. Additionally, there was a significant difference in this parameter between the *A. lappa* cohort (200 mg/kg) and the RA + P cohort (P < 0.01) (Figure 4). 

### 4.5. Impact of Arctium lappa Root Extract on Serum Concentrations of Cytokines of Rheumatoid Arthritis Rats

**Figure 5.**
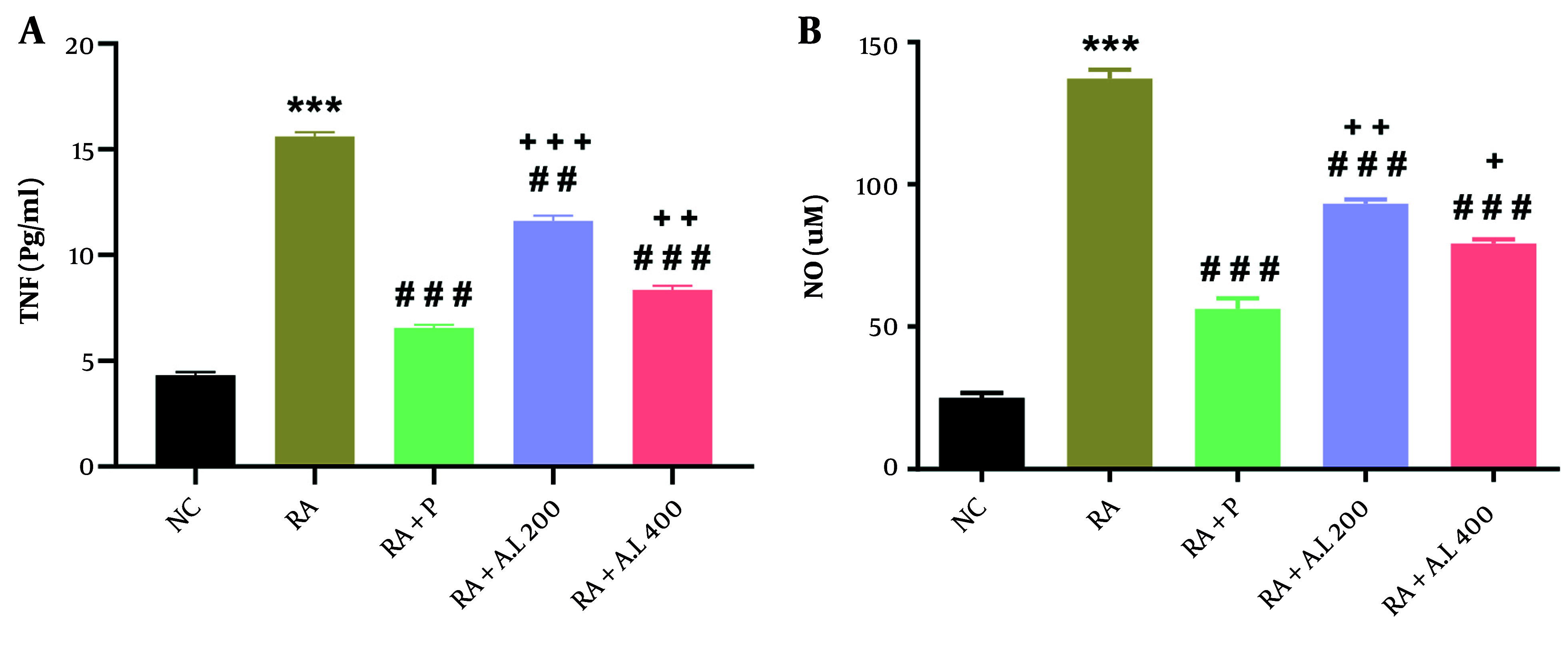
Effect of *Arctium lappa* extract on serum A, tumor necrosis factor-α (TNF-α) and B, nitric oxide (NO) levels in RA-induced rats. Data are presented as mean ± SEM (n = 5). Significant differences were tested by one-way ANOVA followed by Tukey’s post-hoc test; *** P < 0.001 vs. NC group. ## P < 0.01, ### P < 0.001 vs. RA group. + P < 0.05, ++ P < 0.01, +++ P < 0.001 vs. RA + P group. (Abbreviations: NC, normal control; RA, rheumatoid arthritis; P, prednisolone; AL, *Arctium lappa*).

We observed significant differences in serum concentrations of TNF-α [F (4, 20) = 495.4, P < 0.0001; Figure 5A] and NO [F (4, 20) = 281.7, P < 0.0001; Figure 5B] among the cohorts. Post-hoc analysis revealed that RA rats had significantly elevated serum TNF-α and NO concentrations compared to the NC cohort (P < 0.001). Conversely, prednisolone administration significantly reduced these concentrations (P < 0.001). Furthermore, oral treatment with *A. lappa* at doses of 200 and 400 mg/kg significantly reversed these changes, decreasing serum TNF-α (P < 0.01 for 200 mg/kg and P < 0.001 for 400 mg/kg) and NO (P < 0.001) concentrations in a dose-dependent manner. It was also noted that cytokine concentrations in the *A. lappa* cohorts (200 and 400 mg/kg) were higher than those in the RA + P cohort (P < 0.05 to P < 0.001) (Figure 5A and B).

### 4.6. Impact of Arctium lappa Root Extract on Serum Concentrations of Glutathione and Superoxide Dismutase Activity of Rheumatoid Arthritis Rats

**Figure 6.**
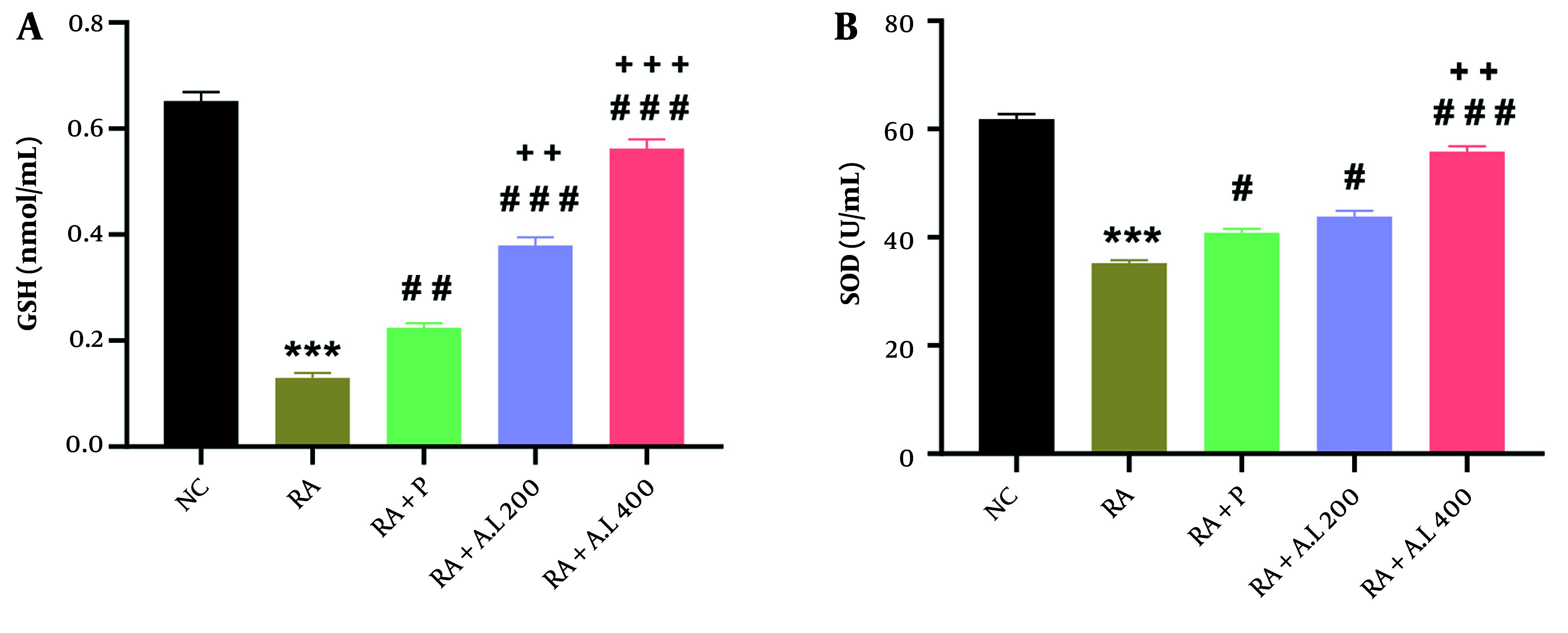
Effect of *Arctium lappa* extract on serum A, glutathione (GSH) levels and B, superoxide dismutase (SOD) activity in RA-induced rats. Data are presented as mean ± SEM (n = 5). Significant differences were tested by one-way ANOVA followed by Tukey’s post-hoc test; *** P < 0.001 vs. NC group. # P < 0.05, ## P < 0.01, ### P < 0.001 vs. RA group. ++ P < 0.01, +++ P < 0.001 vs. RA + P group. (Abbreviations: NC, normal control; RA, rheumatoid arthritis; P, prednisolone; AL, *Arctium lappa*).

One-way ANOVA revealed significant effects of the intervention on GSH concentrations [F (4, 20) = 240.9, P < 0.0001] and SOD activity [F (4, 20) = 151, P < 0.0001]. As shown in Figure 6A, there was a significant decrease in serum GSH concentrations in the RA cohort relative to the NC cohort (P < 0.001). However, these concentrations were significantly increased in prednisolone-treated RA rats (P < 0.01). Treatment with *A. lappa* extract at doses of 200 and 400 mg/kg also increased GSH concentrations considerably compared to the RA cohort (P < 0.001) in a dose-dependent manner. Moreover, *A. lappa* treatment in the RA + A.L. groups was more effective at increasing GSH concentrations compared to the prednisolone treatment cohort (P < 0.01 to P < 0.001) (Figure 6A). 

As demonstrated in Figure 6B, SOD activity was significantly lower (P < 0.001) in the RA cohort relative to the NC cohort. Treatment with prednisolone effectively enhanced SOD activity in the serum of RA rats (P < 0.05). Furthermore, *A. lappa* treatment at 200 and 400 mg/kg improved SOD activity relative to the RA cohort in a dose-dependent manner (P < 0.05 for 200 mg/kg and P < 0.001 for 400 mg/kg) (Figure 6B). Treatment with *A. lappa* at 400 mg/kg was the most effective in increasing SOD activity compared to the RA + P cohort (P < 0.01) (Figure 6B). 

### 4.7. Impact of Arctium lappa Root Extract on the Histopathology of Rheumatoid Arthritis Rats

**Figure 7.**
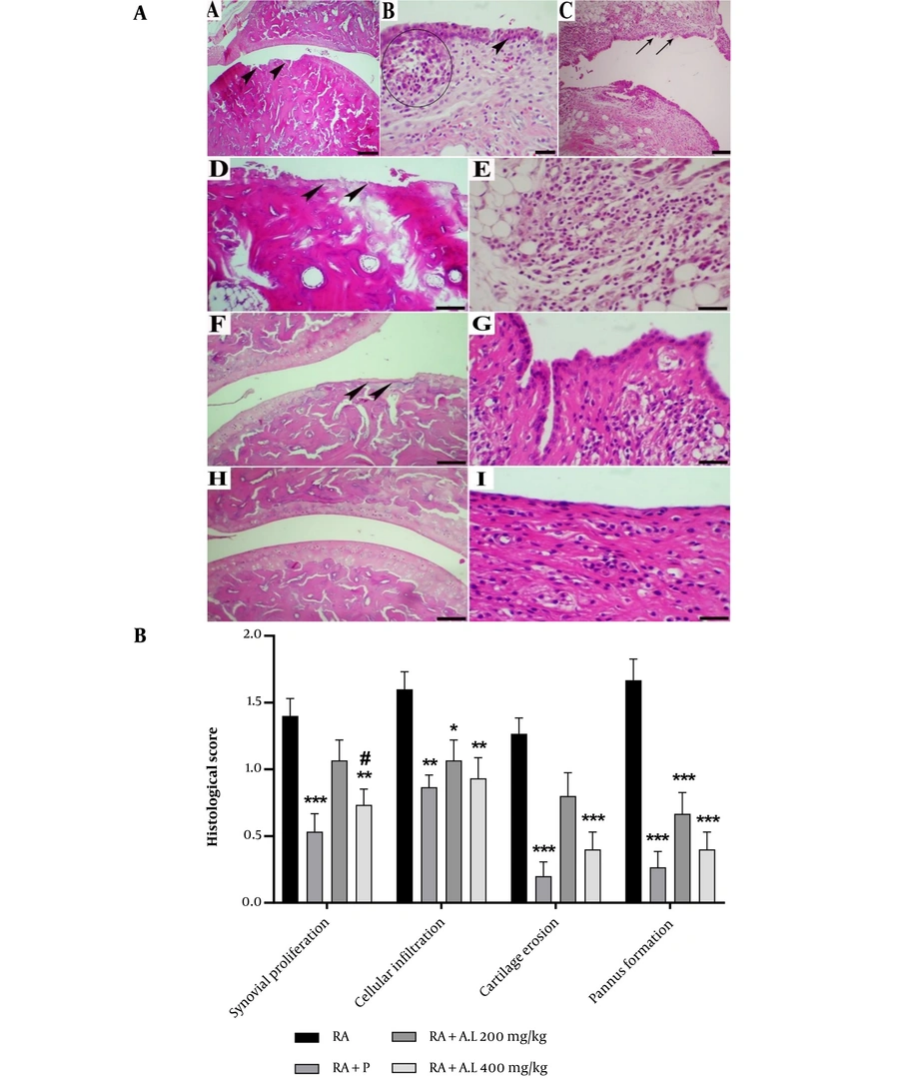
Histopathological lesion in the ankle joint of arthritis control group [hematoxylin and eosin (H&E) staining]. A, Moderate cartilage erosions and necrosis (arrowheads) (scale bar = 150 µm); B, moderate synovial hyperplasia (arrowhead) and pannus formation (circle) (scale bar = 70 µm); C, necrosis of synoviocytes (scale bar = 150 µm). Histopathological lesion in the ankle joint of *Arctium lappa* extracts groups (H&E staining); D, *Arctium lappa* (200 mg/kg), moderate cartilage erosions with degenerated chondrocytes (arrowheads) (scale bar = 70 µm); E, *Arctium lappa* (200 mg/kg), moderate synovial inflammation (scale bar = 70 µm); F, *Arctium lappa* (400 mg/kg), mild cartilage erosions with degenerated chondrocytes (arrowheads) (scale bar = 150 µm); G, *Arctium lappa* (400 mg/kg), mild synovial inflammation (scale bar = 150 µm); H, prednisolone group, normal articular cartilage; I, mild synovial hyperplasia and infiltration of few inflammatory cells (scale bar = 70 µm); B, Histopathological evaluation of ankle joints in different groups. Data are presented as mean ± SEM. * P < 0.05, ** P < 0.01, *** P < 0.001 vs. RA group. # P < 0.05 vs. RA + P group. (Abbreviations: RA, rheumatoid arthritis; P, prednisolone; AL, *Arctium lappa*).

In the microscopic examination of joints, no abnormal structures were observed in the NC rats. Specifically, the cartilage exhibited a smooth articular surface with normal chondrocytes embedded within the typical cartilage matrix, surrounded by an intact synovial membrane. In contrast, the tibiotarsal joints of rats that received CFA displayed mild to severe tissue changes. The central histopathologic lesions included moderate cartilage erosions and necrosis, moderate to severe inflammatory cell infiltration (predominantly mononuclear cells, including lymphocytes and plasma cells) in the connective tissue around the joint, edema, moderate synovial hyperplasia, and pannus formation (Figure 7A). 

The severity of lesions was significantly reduced in the cohorts treated with *A. lappa* extract and prednisolone relative to the arthritis control cohort. Both low and high doses of *A. lappa* mitigated synovial hyperplasia, inflammatory cell infiltration, interstitial edema, and pannus formation in a dose-dependent manner. In the prednisolone cohort, the articular cartilage appeared normal, with some chondrocyte necrosis within the matrix. Mild inflammatory cell infiltration and edema were recorded around the joint, but no pannus formation was observed (Figure 7A). Regarding pathological scores, the lowest score of ankle joint lesions was noted in the prednisolone cohort, followed by the *A. lappa* 400 mg/kg, *A. lappa* 200 mg/kg, and arthritis control cohorts, respectively [F (3, 224) = 42.99, P < 0.0001] (Figure 7B). 

## 5. Discussion

**Figure 8.**
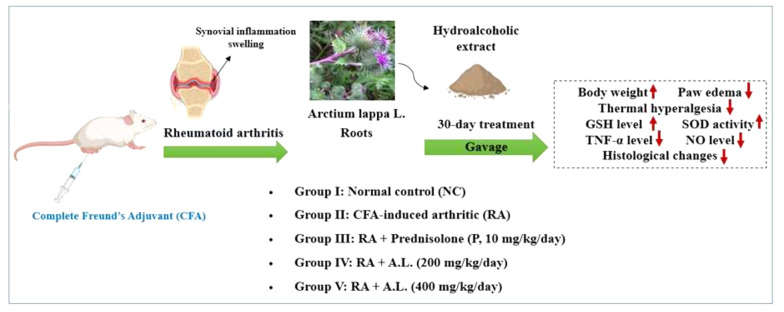
Summary of conclusion

This investigation demonstrated for the first time that oral administration of a hydroalcoholic extract of *A. lappa* root dose-dependently improved joint function and prevented histological changes in rats with RA. The extract reduced inflammatory cytokines and paw volume, recovered antioxidant enzyme concentrations, alleviated thermal hyperalgesia, and maintained body weight (Figure 8). 

Previous investigations have documented that RA is a progressive autoimmune disorder that primarily affects the synovial membrane, leading to the gradual destruction of both cartilage and bone, as well as various clinical implications such as malignancy, cardiovascular disorders, psychiatric issues, and lung diseases (30, 31). Researchers have identified that the immune system and inflammatory cytokines play crucial roles in the pathogenesis of RA (32). Elevated pro-inflammatory cytokine expression (TNF, NO) damages synovial tissues, causes vasodilation at the site of edema, and exacerbates the inflammatory response in the synovial fluid of RA patients (33-35). Therefore, these cytokines are effective targets for the treatment of inflammatory diseases.

On the other hand, oxidative stress plays a notable role in the pathogenicity of oxygen radicals, contributing to joint tissue damage and chronic inflammation in patients with RA (10, 36). Research has shown that TNF-α can induce higher concentrations of oxidative stress, further amplifying tissue destruction and periarticular deformities (37, 38). Increasing evidence has also highlighted the beneficial effects of herbal medicines and alternative interventions on the symptoms of RA (39, 40).

In the current study, we utilized an adjuvant-triggered arthritic animal model and observed the manifestation of RA disease parameters (41). *Arctium lappa* root treatment significantly mitigated paw volume and enhanced body weight and thermal hyperalgesia threshold relative to RA rats, though the effect was less than that of prednisolone. Moreover, *A. lappa* suppressed inflammatory mediators and restored the histopathology of the joint and antioxidant defenses relative to RA rats. To our knowledge, there are limited investigations on the antioxidant and anti-inflammatory effects of *A. lappa* extract in various models. Alhusaini et al. applied *A. lappa* root extract to lead (Pb)-induced liver injury in a rat model and found it acted as an antioxidant and anti-inflammatory agent with hepato-protective effects (42). Maghsoumi-Norouzabad et al. demonstrated that daily consumption of *A. lappa* root tea (2 g/150 mL water) for 42 days decreased serum concentrations of IL-6, CRP, MDA, and enhanced GSH concentrations and SOD activity in patients with knee osteoarthritis (43). Another study found that oral administration of *A. lappa* hydroalcoholic extract markedly suppressed neutrophil formation and reduced the inflammatory status (IL-6, TNF-α, and NO concentrations) in mice with lipopolysaccharide (LPS)-triggered inflammation (18). Additionally, the antioxidants and anti-inflammatory effects have been observed using a similar herbal extract in arthritis models (44-46).

The anti-inflammatory and antioxidant properties of *A. lappa* are closely linked to its bioactive components. For example, lignan compounds such as arctigenin (47) and arctiin (48) from *A. lappa* seeds have demonstrated anti-inflammatory effects by inhibiting iNOS expression and suppressing the NF-κB pathway, leading to decreased concentrations of TNF-α and IL-1β in LPS-activated macrophages. Additionally, the quercetin compound from *A. lappa* leaves has been demonstrated to restore the activity of antioxidant enzymes (SOD, CAT, and GSH-Px) and reduce concentrations of ROS and TBARS in macrophage cells (49). Therefore, the *A. lappa* L. root extract may help prevent arthritis and joint destruction in arthritis-triggered animals by regulating inflammation and oxidative stress.

### 5.1. Conclusions

Taken together, the findings of this study indicate that the hydroalcoholic extract of *A. lappa* root exhibits notable anti-arthritic activity by inhibiting the release of inflammatory cytokines and enhancing antioxidant concentrations in CFA-triggered arthritis rats. However, further research is necessary to elucidate its molecular mechanisms and potential clinical applications.

### 5.2. Limitations of the Study

Based on the results of this study, the following suggestions are made: Study the effect of *A. lappa* on signaling pathways (e.g., NF-κB, Nrf2) and assess experimental tests that evaluate chronic arthritic pain, such as the von Frey test.

## Data Availability

The dataset presented in the study is available on request from the corresponding author during submission or after publication.
